# Effects of Shaving

**Published:** 1855-03

**Authors:** E. Sanborn

**Affiliations:** Andover, Mass.


### EVIL EFFECTS OF SHAVING THE BEARD.

BY E. SANBORN, M. D., ANDOVER, MASS.

I am prompted, by remarks recently made by a learned professor of theology, on his conflicts with the razor, to ask if the habit of shaving is not both deleterious to the physical health, and detoriorating to the races which practise it? The professor thinks that by shaving daily for forty years he has wasted a year and a half of laboring time, and nearly fifty pounds of beard, to say nothing of the blood and tears he has shed, and the agony he has endured. It has, he says, been the great misery and *waste-way* of his life, and he would give the world if he possessed it, as would thousands of his brethren, if custom would sanction the omission of shaving.

Now I have, within the past year, perhaps owing to my own long-beardedness, which, by-the-by, is indispensible, heard so many similiar remarks, both from eminent ministers of the gospel and other citizens of most sterling sense, and witnessed personally so much evil from the habit, that I cannot forbear saying, that comparing the present with the past, there seems to me a fearful deterioration in the physical organization of our race, worthy of of the most serious and immediate attention of every true-hearted American, patriot and philanthropist. It cannot but be obvious to all who regard the future defences of our country, that large families of robust and healthy children are far less numerous now than they were in former days. Scrofula, that truly direful scourge of pro-shaving England and America invades, in some one or more of its hideous forms, almost every domestic circle of our American population. The indescribable pangs of neuralgia, which were scarcely known to our ancestors, are now as familiar as household words in our families. Nearly as much is true of bronchial and catarrhal affections, erysipelas, heart-disease, permature defection of the senses, physical deformity and prostration; coughs, and consumptions which waste away the strength and beauty of our homes, and dry up the fountains of our joy. Another eminent professor, whose active love of humanity has familiarized him with the customs and conditions of many nations says—" I have been much among the Abrahamic race who never violate the command—not to mar even the corners of their beards, and I never saw an instance of drunkenness or pauperism, nor of permature physical debility or hereditary disease, as they exist among the pro-shaving nations. Who shall say that this chosen people, this most heavily bearded of all races, is not by their religious devotion to the laws of health, destined to stand upon the earth and fill it with unabridged, unadulterated manliness, when other nations of greater boasted light and knowledge shall, by their fool-hardy violations of nature, have consumed themselves and passed away?"

The aboriginal inhabitants of our soil and climate were once brave, powerful, and numberless almost as "the stars in the sky, the leaves on the trees, and the sands on the sea-shore." But they waged war unceasingly against nature. They resisted her kindly efforts to mantle their faces with manly beards, grew each generation more and more effeminate, because an easy prey to their enemies, and now, like the beard which they so obstinately uprooted from their faces, they are themselves uprooted from the face of the earth. The Chinese, too, have been "*shorn of their locks*" and their strength, till as a nation they have but little more than a nominal existence. I speak from the experience and close observation of more than twenty years of dental practice, in saying that I have not a doubt that to the loss of nutrition, and to the exposure and derangement of the animal functions caused by incessantly scraping off the beard, is to be attributed much of the alarming increase of premature defection in the dental organism, which tends directly to imperfect mastication, indigestion, dyspepsia, and "all the ills that flesh is heir to." It is no uncommon thing for children 10 or 12 years old to need extensive operations on their secondary teeth, nor young men and women of 20 to require whole sets. Truly, with a vengeance have the fathers eaten their sour grapes. At this rate, how soon as a nation shall we be "*sans* eyes, *sans* teeth, *sans* everything?"

This increasing degeneracy is no new idea; but while one attributes it to a variable climate, another to unventilated houses, another to lead poisoned water, and others to flour *too finely*, or to food too coldly, too hotly, or too hastily *bolted*, few only venture to speak boldly of the tons and tons of the physical stamina and manliness of pure Americanism which is daily sacrificed by the relentless razor to that despotic Delilah, Fashion. Pulmonary disease, bronchial inflammation, cough and night sweats, had so reduced my system more than a year ago, that eminent physicians in Boston and elsewhere assured me that my existence would soon be terminated. Resolved, however, if go I must, to go as whole and resistingly as possible, I discarded at once, and as I now joyfully believe *forever*, the use of my razor. The result is—a restoration to almost perfect health, and freedom from thirstiness and debility which seemed formerly to demand so much medication and artificial stimulation. That I have a grizzly beard is most true, and that it is offensive to existing tastes it grieves me to say is quite as true. But that it is repulsive, excessively so, to the shallow-brained dupes and votaries of mere unsanctified custom and conventionality, troubles me so little that I survive the caustic joke, the scornful smile, the withering frown, the cold shoulder and diminished patronage, assured that I am a thousand fold repaid not only in restoration to health, but in "a soul's calm sunshine and a heart felt joy" which exist only in a consciousness of rectitude that can afford to be laughed at.

It is far from self-conceit which prompts these remarks, or urges me to say that the accumulation of facts which as a matter of course must have resulted from my long-bearded experience, will enable me to give comforting suggestions to such as are almost persuaded to follow the teachings of nature and true philosophy in this matter. The task is harder in later than in earlier life, before the beard is excited to unnatural growth. And as there is, as I most solemnly believe, not one well-founded argument why men of any age or condition should continue the habit of shaving, there can be none why boys should ever commence it. Nor would one of a thousand of them do so, if they only knew its evil consequences. It not only destroys the germs of their future physical health and manly beauty, but it wastes away the dews of their youth, their native simplicity, their truthfulness and confidence in the wisdom of their Creator. Neither the image of God nor any of his works remain sacred in their sight.

The habit of shaving is not of " Origin Divine " as thousands seem to think, but quite the reverse. The ancient patriarchs, the holy prophets, Christ and h[illegible]s disciples, and the earlier and probably purer christians, deemed it a violation of the laws of their nature. Alexander enforced it upon his army that they might thereby gain a bloodier conquest. The nobility of Spain adopted it through courtesy to their beardless prince. The mass were of course subjected to the humiliating process, but expressed their repugnance to the outrage in the well-known proverb :—"Since we have lost our beards, we have lost our souls"—that is, ourselves, our identity. We are rather soulless slaves, than the men our Maker made and designed us to be.

Will not the free-born sons of America, whose pure patriotism scorns the dictation of foreign potentates, dare to be morally as well as politically free—free from all conventionalities which oppress common humanity, and weigh heavily on the mass of our population; and especially free from every influence which insidiously tends to vitiate and depress the true manliness of man, and womanizes those masculine and gigantic powers which are to be our country's defense against the jealous, hungry, couching nations about us? And will not their mothers, sisters, wives and daughters, second these efforts, and exercise their own good taste in creating and sustaining such purely American fashions and habits as will, to the end of time, render them and their progeny still more excellent in all the various relations of life?—*Boston Med. and Surg. Jour.*

